# Blood-brain-barrier modeling with tissue chips for research applications in space and on Earth

**DOI:** 10.3389/frspt.2023.1176943

**Published:** 2023-08-09

**Authors:** Anne Yau, Aditi Jogdand, Yupeng Chen

**Affiliations:** Nanomedicine Lab, Department of Biomedical Engineering, University of Connecticut, Storrs, CT, United States

**Keywords:** tissue chip, blood brain barrier, nanomaterials, regenerative medicine, tissue engineering

## Abstract

Tissue chip technology has revolutionized biomedical applications and the medical science field for the past few decades. Currently, tissue chips are one of the most powerful research tools aiding in *in vitro* work to accurately predict the outcome of studies when compared to monolayer two-dimensional (2D) cell cultures. While 2D cell cultures held prominence for a long time, their lack of biomimicry has resulted in a transition to 3D cell cultures, including tissue chips technology, to overcome the discrepancies often seen in *in vitro* studies. Due to their wide range of applications, different organ systems have been studied over the years, one of which is the blood brain barrier (BBB) which is discussed in this review. The BBB is an incredible protective unit of the body, keeping out pathogens from entering the brain through vasculature. However, there are some microbes and certain diseases that disrupt the function of this barrier which can lead to detrimental outcomes. Over the past few years, various designs of the BBB have been proposed and modeled to study drug delivery and disease modeling on Earth. More recently, researchers have started to utilize tissue chips in space to study the effects of microgravity on human health. BBB tissue chips in space can be a tool to understand function mechanisms and therapeutics. This review addresses the limitations of monolayer cell culture which could be overcome with utilizing tissue chips technology. Current BBB models on Earth and how they are fabricated as well as what influences the BBB cell culture in tissue chips are discussed. Then, this article reviews how application of these technologies together with incorporating biosensors in space would be beneficial to help in predicting a more accurate physiological response in specific tissue or organ chips. Finally, the current platforms used in space and some solutions to overcome some shortcomings for future BBB tissue chip research are also discussed.

## 1 Introduction

In the past few decades, tissue chips have been developed and used by researchers to understand many of the complex problems and phenomena that need to be answered. Unlike 2D cell cultures on a tissue culture plate, tissue chips allow researchers to determine various parameters such as tissue chips fabrication materials, types of hydrogels used, shear stress in tissue chips, cell types used, and biomechanical stimuli on tissue chips to accurately mimic the human body, allowing for the study of the cell’s reaction to things such as drugs and mechanical stimuli (Low and Tagle, 2016). Tissue chips have become common tools for research, and they come in different forms and sizes. They are powerful tools as they are a bridge between modern tissue engineering and the power of computers and imaging (Donoghue et al., 2021). Due to the complexity of the human body, it is difficult to capture details in *in vitro* studies in a monolayer cell culture environment, therefore allowing researchers to take one step further into conducting research on varied processes to obtain accurate and precise results. Tissue chips can be designed in a way where researchers decide on the parameter with the use of computer software and programs to determine fluid flow or pressure, which is a limitation found in conventional *in vitro* experiments (Low and Tagle, 2016).

Tissue chips have been established for various organs and parts of the body, such as the study of blood brain barrier (BBB) in both 2D and three dimensional (3D) environments which is discussed in this review paper. BBB is a highly specialized barrier that separates the blood vessels in the brain from the brain tissue. It is formed by a layer of tightly-packed cells called endothelial cells, which line the walls of the brain’s capillaries. The BBB is responsible for protecting the brain from harmful substances and regulation of molecules, like water and blood-dissolved gasses across the blood-brain interfaces (Seo et al., 2020). Studying the BBB in space is important because space travel can have significant effects on the human body, including the nervous system. Understanding how BBB functions in space can provide additional information into neurological changes that astronauts experience during prolonged space missions, by identifying potential risks and developing strategies to mitigate the risks, ensuring safety of astronauts. Furthermore, BBB plays a crucial role in maintaining homeostasis of the brain by regulating the exchange of substances between the bloodstream and the brain (Knox et al., 2022). Changes in the microgravity environment may alter the BBB’s structure and function, potentially affecting brain health and cognitive performance. Studying BBB in space can further provide insights on how these changes occur and explore potential countermeasures to protect the brain health and optimize cognitive function during space missions.

**FIGURE 1:**
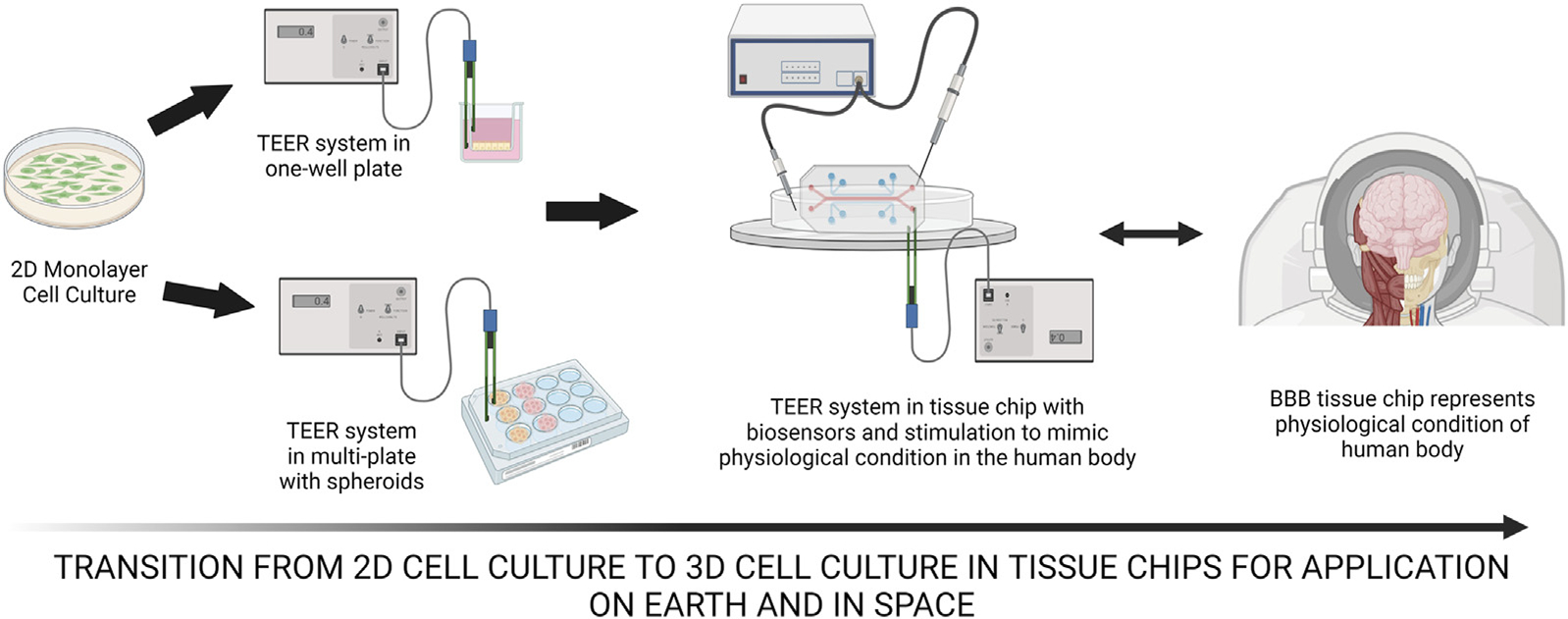
The transition of 2D monolayer cell culture to TEER system in one-well plate then to possibly TEER systems in a plate with spheroids to test for resistance of spheroid culture. BBB tissue chips can be utilized both on Earth and in space with the incorporation of hydrogels, scaffolds, biosensors, biomechanical stimulation and others to observe and understand the mechanism of the human physiological system. Created with BioRender.com.

In 2016, as a part of the Tissue Chips for Drug Screening program, National Center for Advancing Translational Sciences (NCATS), and the International Space Station (ISS) National Laboratory partnered up to fund a Tissue Chips in Space Initiative to utilize the advancing technology of tissue chips to study human diseases in the unique microgravity environment in space (Low and Giulianotti, 2019). Microgravity has been shown to play a role in differentiation and specialization of cells (Shi et al., 2017; Grimm et al., 2020). These changes occur due to the abrupt fluid flow differences as well as other space stressors. Spaceflight and microgravity have been linked to a weaker immune system (Crucian et al., 2018). Many immune cell types are blocked and secretion of cytokines changes, leading to immunosuppression (Grimm et al., 2016). This is an important factor when studying the mechanisms of the BBB, acting as a protective barrier surrounded by immune cells like microglia. Microgravity has been shown to have a tremendous impact on cell behavior where the cells aggregate and an increase of apoptotic cells are observed (Masiello et al., 2014; Low and Giulianotti, 2019; Prasad et al., 2020). It has also been shown to accelerate the time required to reach a disease state (Yau et al., 2023). Biolo et al. (2003) were able to describe the commonalities between space-related physiological change and aging. They found that the two mechanisms that contributed to bone demineralization in elderly people were also observed in male astronauts, specifically when there was a decreased mechanical load and decreased hormone levels. Microgravity’s impact can be a powerful tool when doing research about the blood brain barrier, for both disease modeling and drug screening, which can be effectively studied with the help of tissue chips. This article reviews the transition of 2D BBB models and how they can be applied tissue chip models and discusses the integrity of BBB *in vivo* in comparison to microfluidic systems. The influences affecting BBB on Earth and in space, the implications of tissue chips on Earth and in space, as well as the integration of biosensors and current tissue chips in space are discussed seen in Figure 1.

## 2 The transition and translation of 2D models to microfluidic models

2D cell culture systems have been extensively studied and are still used among the researchers. In these systems, cells grow on flat dishes, made from plastic. They are inexpensive and well-established and have a substantial body of literature dedicated to studying and utilizing them. Many researchers are still using this method for their experiments because they are 1) low-cost and efficient (Duval et al., 2017; Kapalczynska et al., 2018) such as the use of Transwell system like the Ready-To-Use BBB KitTM, that’s commercially available from PharmaCo-Cell Company (Tokyo, Japan) (Thomas et al., 2017), 2) mainly used for preliminary studies and high throughput drug screening in a research and development settings (Antoni et al., 2015; Breslin and O’Driscoll, 2013) and 3) are required by the Federal Drug Administration (FDA) when testing for drug development (FDA, 2018; FDA, 2021). Since cells are growing in a monolayer, 2D systems allow for everything to have equal access to media and nutrients (Duval et al., 2017). Different types of *in vitro* BBB models have been developed and introduced in the past few decades, like the Transwell system, microfluidic chip systems with porous membranes, spheroid-based approach and hydrogel-laden microfluidic chip systems (Seo et al., 2020). The main models used for the BBB specifically are Transwell Co-Culture Systems (Stone et al., 2019; Williams-Medina et al., 2020). They are easy-to-use devices which allow for the user to insert the variety for cell types present in the *in vivo* BBB. Transwells are widely used because they are easy to use and less expensive than building a new model. They come in mono-, co-, tri-culture systems (Wolff et al., 2015). Monocultures are the most basic form, and they rely solely on endothelial cells. Hawkins et al. (2006) showed in their research that a functioning BBB can be built without astrocytes.

One major obstacle in the study of the brain is overcoming the low permeability of the BBB. The BBB acts as a structural and functional gate for microorganisms, drugs, and ions. It tightly regulates what is allowed into the brain with the help of endothelial cells, pericytes, and astrocytes. Since the BBB is one of the most selective barriers in the human body, it is important that its selectivity be included in any models. The trans-endothelial electrical resistance (TEER) is a common non-invasive method used to determine permeability measurements within the BBB complex protective network (Srinivasan et al., 2015). Tight junctions are an essential component of a fully functioning BBB. TEER measurements are an essential tool for assessing biomarkers of the BBB and its regulation in health and disease, measuring the barrier permeability and allowing the researchers to capitulate their design to the actual BBB. Cocultures including endothelial cells and astrocytes (Abbott et al., 2012) or pericytes in a co-cultured BBB Transwell model were used to study the transport of amyloid beta plaque between the blood and the brain and the impact of pericytes (Candela et al., 2015). In Booth and Kim (2012), The TEER values in a microfluidic dynamic model have been found to be significantly higher than a static model. With the addition of shear stresses and fluid flow, Cucullo et al. (2008) also saw a 20 time increase in TEER for the dynamic model compared to a static BBB, to show that TEER is a vital part of BBB function analysis *in vitro* studies (Cucullo et al., 2008; Liang, 2021).

A TEER *in vitro* test can give us important information on the status of the model. For a normal BBB, the values of TEER may be in a range between 1,500 and 8,000 Ω cm^2^, and values of 150–200 Ω cm^2^ are the lower limit (Reichel et al., 2003; Lochhead et al., 2020; Fangchao Yin et al., 2022). With TEER measurements, a set of electrochemical sensors are used to measure the ability of a biological barrier to impede the flow of electrons across it. On the other hand, Lyu et al. (2021) performed their permeability tests with fluorescently labeled dextran in their tissue chips and found that the use of TEER allows for more reproducible results. Their studies showed that TEER measurement in tissue chips or in conventional BBB cell culture methods are essential in the understanding of barrier permeability. In order for a study on the BBB to provide fruitful information especially for space-related applications, studies on utilizing TEER for the *in vivo* system, such as Srinivasan’s group utilizing microelectrodes and able theory in rats, must be researched further design their own tissue chips layouts to fit the goal their study addresses (Srinivasan et al., 2015). Another way to study the integrity of BBB *in vivo*, radiolabeled tracers such as radioactive isotopes or contrast agents, can be injected into the bloodstream. The presence and the amount of tracers in the brain tissue can assess the integrity of BBB, with the use of various imaging techniques like positron emission tomography (PET) or single-photon emission computed tomography (SPECT).

In addition to being the barrier acting as a gate regulating substances in and out of the brain, the BBB works in tandem with the immune system to protect one of our most important organs (Ballabh et al., 2004). Preservation of natural shape can be pertinent to a study about the BBB and if drugs cause any changes to structure that contribute to changes in permeability. Different markers and plasma proteins of varying molecular weights (MW) are employed to assess the permeability of the blood-brain barrier (BBB). Furthermore, alterations in the expression of tight-junction proteins serve as the foundation for the BBB’s structural integrity, and various imaging techniques are utilized to investigate BBB disruption (Sun et al., 2021). In the protocol by Hajal et al., BBB microvascular networks is developed for studying molecular permeability. The methods are versatile and applicable to various research areas. Additionally, the flexibility of this protocol extends to other microvascular networks and *in vitro* vascular models, including the study of tumor metastasis, local permeability changes, and flow-induced effects on endothelial function (Bischoff et al., 2016). Current BBB *in vitro* models can enable further the study of the cellular and molecular mechanisms in BBB but there is a lack of models that combine fast and high-throughput readouts with relevant conditions, found in the *in vivo* conditions (Wevers et al., 2018). Furthermore, cells in monolayer cell culture lack the modulation by neighboring cells as well as mechanical stimuli that are natural, like shear stress in native environment. Enhanced cell communication and integration in 3D cultures enable the development of barrier tissues, vital for organ survival and compartmentalization. 3D cultures provide a better representation of how cells respond to mechanical stimuli, allowing for proliferation and expansion in all directions. In cancer research, certain immune checkpoint proteins exhibit different expressions in 2D models compared to *in vivo*. 3D cultures, such as spheroids and organoids, offer more realistic tumor models. Although 3D cell cultures are more costly compared to typical 2D cultures, they reduce the requirement for animal experimentation due to their enhanced physiological similarity to *in vivo* conditions (Liu et al., 2018; Jensen and Teng, 2020; Yau et al., 2021).

Many researchers have transitioned away from the monolayer cell culture due to the difficulty in maintaining BBB properties over time, where there is a lack of automated waste removal as performed in vivo, naturally. The use of BBB tissue chips allows researchers to study the BBB in a more physiologically relevant way to overcome the limitations of rigid surfaces found in 2D Transwell insert *in vitro* where there are no direct cell-cell interactions and lack of physiological mechanical forces such as shear stress while preserving natural cell shape and grow in various ways or aggregates to form spheroid (Vatine et al., 2019). In addition to that, the utilization of the BBB tissue chip models can enhance researchers with the ability to incorporate multiple cell types in one device. Using tissue chips, researchers could determine BBB integrity in the tissue chip with permeability assays by introducing dextran into the flow such as the ones seen in lung on a chip developed by Frost et al. (2019). By observing a tissue chip with diverse cell types, in relevant physiological conditions, researchers can examine the interactions between microglia and other neural cells once the cells have reached a mature state. Cells can be fluorescently tagged, and therefore can be observed in real-time in microscopy to examine the spatial arrangement of cellular components within the BBB tissue chip (Yau et al., 2019). This allows researchers to observe the organization and distribution of tight junction proteins and other relevant markers. As cells proliferate and differentiate, they undergo changes such as increased oxidative stress, protein accumulation, reduced toxin clearance, and alterations in signaling pathways (Mattson and Magnus, 2006). BBB tissue chips provide a valuable platform for studying the BBB due to their ability to recreate the complex microenvironment and physiological conditions of the BBB in a controlled and scalable manner with translational relevance.

The study of BBB in space is a complex and challenging area of research that has not received as much attention as other aspects of space exploration and human health. Several factors do contribute to the limited focus on studying the BBB in space. One of the factors studying BBB received less research attention because of other research areas that received more attention and resources due to their immediate impact on the astronauts’ health and mission success. As a result, the BBB, being part of the neuroscience fields, may not always be a top priority for research institutions and implementation partners (Mu et al., 2022). In addition to that, technological limitations requiring advanced setup, including automated microfluidic systems, and sophisticated imaging due to the challenging and costly development and deployment of such tools in space. Certain hardware have been developed where it encompassed all-in-one features such as automated waste removal and timely and accurate imaging sessions, these hardware are still lacking in terms of reproducibility and scalability. The manufacturing of cells or conducting high-throughput studies using cell cultures in microfluidic systems is currently considered challenging, but advancements in this field suggest that it may soon become feasible. While the study of the BBB in space may currently face challenges and limited attention, it is important to recognize that scientific priorities and research trends evolve over time. As space exploration expands, and as the potential long-term effects of space travel on the brain become more prominent, there may be increased recognition of the importance of studying the BBB in space. Continued efforts to raise awareness, foster interdisciplinary collaborations, and secure funding may also help address the current gaps and ensure that the BBB receives the attention it deserves in space research.

## 3 Influences that affect BBB tissue chips in space and on Earth

**FIGURE 2:**
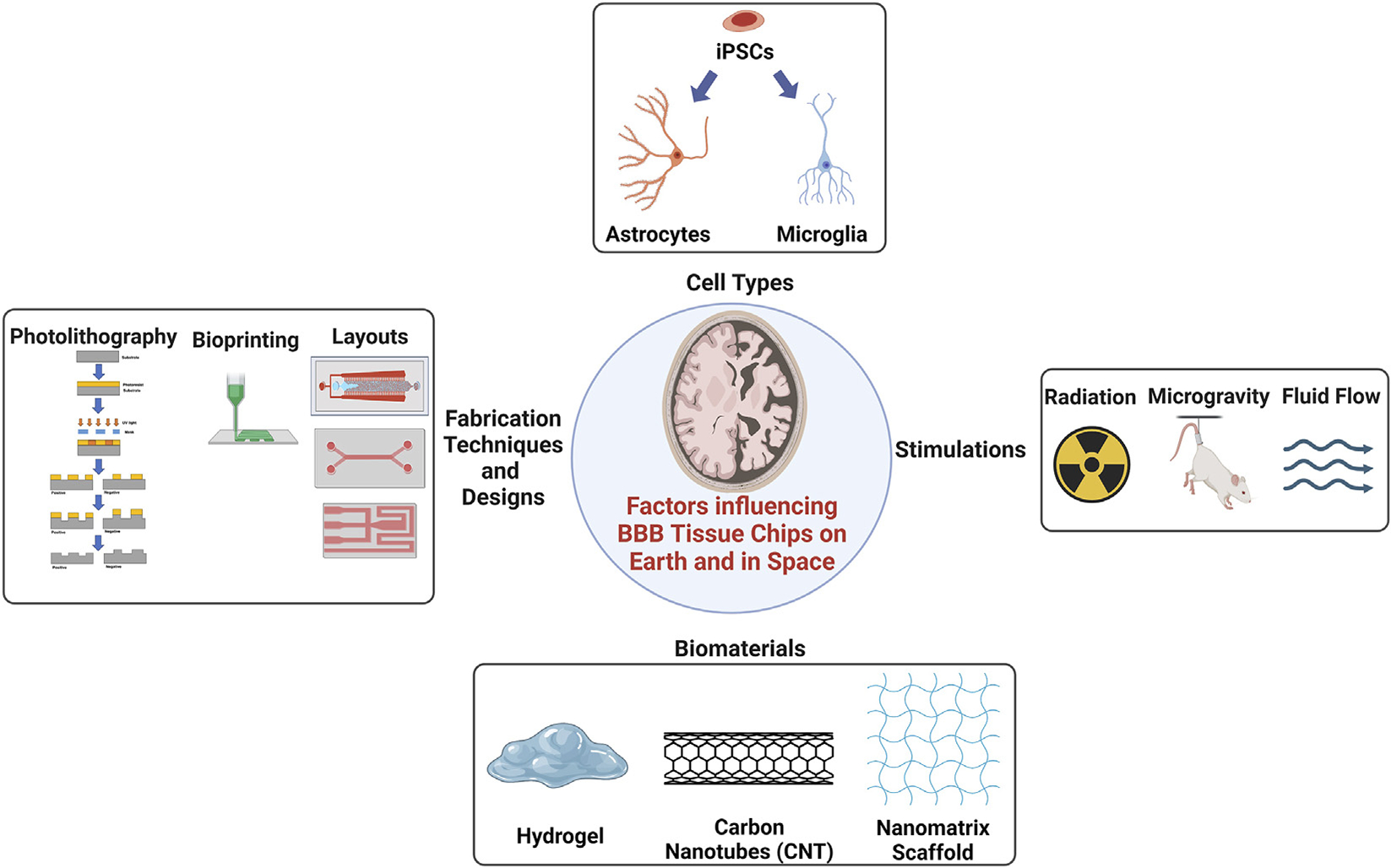
There are many influences affecting cell bioactivity cultured on Earth and in space. The use of cell types, especially the induced pluripotent stem cells (iPSCs) that have the potential to differentiate into any cell types found in the body with adequate nutrition. Simulations to different cell types such as radiation, microgravity, or fluid flow to affect the cells and tissue in the BBB tissue chips. Accumulations of cytokines in tissue chips may occur due to a long-term culture or after radiation exposure which can desensitize the accuracy of biosensors. The use of hydrogels is popular but may not be advantageous in space due to lack of adhesion sites compared to solid scaffolds. The use of CNTs is popular as well but the toxicity of CNTs are still in questions. The use of JBNt from Dr. Yupeng Chen’s lab (Griger et al., 2022; Zhou et al., 2020a; Zhou et al., 2020b) is more favorable due to its injectability and adhesive properties that can aid in cell attachment and encourage proliferation and differentiation in microgravity. Created with BioRender.com.

The BBB tissue chip is an engineered microenvironment that allows for real-time monitoring of cellular responses in various conditions, providing a valuable platform for studying the intricacies of the BBB in a controlled and physiologically relevant manner. It is a bridge between 3D cell culture models of the BBB and advanced computer technologies. The effect of biomaterials used on Earth may differ from the effects of biomaterials used in space. Selecting types of cells for BBB tissue chips is as important as studying the effects of fluidic dynamics and nutrient supply in microgravity. Performing BBB tissue chip experiments in space further introduces additional factors that can affect the tissue chips, including microgravity and radiation. Understanding the influences that affect BBB tissue chips on Earth and in space is critical for optimizing their design, ensuring accurate representation of the BBB, and enabling meaningful investigations into BBB physiology, disease modeling, and drug development, ultimately advancing our understanding of the brain and supporting the health and wellbeing of astronauts and the general population alike. In this section we will discuss the influences affecting BBB tissue chips in space and on Earth as seen in Figure 2. Then, in the next section a few limitations that are currently faced by researchers on Earth in the making of tissue chips, and additional challenges that can be encountered if performed in space are discussed (Chen et al., 2011).

### 3.1 Biomaterials

Hydrogels have been becoming popular in microfluidic manufacturing. They have unique properties, such as high-water content and biocompatibility, making them suitable for various biomedical applications, including tissue engineering or in tissue chips. There are many types of hydrogels, including alginate, gelatin, fibrin, hyaluronic acid, and agarose (Zhao et al., 2020). In space, they can be used to support growth and differentiation. Hydrogels can be engineered to mimic the extracellular matrix (ECM) and provide essential cues for cell behavior and tissue regeneration. Hydrogels can also serve as scaffolds for endothelial cells, astrocytes, and other relevant cell types that comprise the BBB to replicate complex cellular interactions and barrier properties to provide a platform to study drug transport, barrier integrity, and responses to various stimuli (Sood et al., 2022). Wu et al. (2020) mimicked the BBB with a microfluidic chip using fibrin hydrogel holding the brain cells to understand the toxicity of Nε-(carboxymethyl)lysine (CML), a common substance found in food. They found that the CML permeates into the *in vitro* fibrin BBB model causing an increased reactive oxidative species (ROS) levels and inflammatory responses, similar to reported *in vivo* responses. Ayuso et al. (2019) manufactured collagen gel embedded with cells to study a pseudopalisade hypercellular zone in a glioblastoma. In another group, Jeong Kim et al. used collagen hydrogel and 3D printing to assemble a 3D model of the brain’s vasculature.

The mechanical properties of hydrogels are comparable to the ECM, and they are typically injectable. However, hydrogels have poor mechanical properties compared to those of solid scaffolds. Nanofibers are another example of a solid scaffold. Nanofibrous scaffolds provide a high surface area for cell attachment, proliferation, and tissue regeneration, leading to improved tissue integration and functionality (Rohde et al., 2022). They can be used as substrates to engineer various tissues and organs, including bone, cartilage, and blood vessels. With a wide range of applications, nanofibers can be fabricated with many different processes, including electrospinning and or self-assembly. They are made with biopolymers and polysaccharides, which gives them low toxicity (Torres-Martinez et al., 2018; Priya et al., 2022). Carbon nanotubes (CNT) have gained significant attention in the field of biomedical applications due to their unique properties and potential benefits. They have a high surface area to volume ratio and might have antimicrobial properties (Zhengping Zhou et al., 2009; Torres-Martinez et al., 2018). They also have high mechanical strength, stiffness, and excellent electrical and thermal conductivities (Zhengping Zhou et al., 2009; Lee et al., 2021). CNTs have been shown to enhance neuronal excitability because of their conductivity to establish the cell-cell connection, creating efficient neural pathways. Moreover, CNTs have demonstrated inherent therapeutic effects in preventing strokes *in vivo* (Lee et al., 2011). Despite the potential benefits of carbon nanotubes, it is important to note that their biocompatibility, toxicity, and long-term effects are still subjects of ongoing research (Murjani et al., 2022). They are found to be toxic when they are absorbed into the intracellular space due to poor dispersion and formation of aggregates (Griger et al., 2022). Proper functionalization, purification, and characterization methods are crucial to ensure their safe use in biomedical applications. Regulatory considerations and ethical aspects also need to be taken into account when translating CNT-based technologies into clinical practice.

Dr. Yupeng Chen’s group developed a deoxyribonucleic acid (DNA)-inspired nanomaterial to overcome the limitations of both hydrogel and solid scaffold such as CNTs. These DNA-inspired nanomaterials developed by Chen et al. are derived from DNA-molecules adenosine and thymine, contributing to their biocompatibility, where they self-assemble to form the Janus base nanotubes (JBNt). These JBNts can be used for different applications such as drug delivery or as scaffold for tissue engineering after a second self-assembly process (Zhou et al., 2020a; Lee et al., 2021). JBNt are nanometers in diameter but can self-assemble into a long nanotube to morphologically mimic collagen scaffold. In 2019, Chen *et. al*. has been using JBNt to deliver RNA therapeutics into cells because they are small enough to enter the cells after sonication, forming Janus base nanopiece (JBNp). In this case, the JBNp can be used to deliver drugs crossing the BBB tight junctions due to its small size and biocompatibility. On the other hand, JBNt can self-assemble with various ECM proteins, forming the Janus base nanomatrix (JBNm). These JBNms are injectable and yet provide a solid adhesion site for cell attachment and proliferation unlike hydrogel and solid scaffold (Zhou et al., 2020b; Zhou et al., 2021; Landolina et al., 2022). With JBNm, the integrity of the barrier may be strengthened due to the adhesion site provided by the DNA nanomaterials. These nanomaterials can be proven to be helpful when performed in space because they are injectable and biocompatible like hydrogel and still provide an adhesion site for cell attachment, similar to solid scaffold, which is preferable for cell activity. The use of both JBNp and JBNm can prove to be useful applications injected into tissue chips both on Earth and in space.

### 3.2 Cell types

Understanding the BBB’s intricate mechanisms requiring its dysfunction in diseases like neurodegenerative disorders and brain tumors requires sophisticated research tools. In recent years, microfluidic devices have emerged as valuable platforms for studying the BBB in a controlled and physiologically relevant manner especially in microfluidic systems. These devices enable researchers to mimic the structure and function of the BBB, providing insights into its physiological properties and the interactions between different cell types involved in maintaining its integrity. Other than TEER method, the integrity of the tight junctions of the BBB can be determined using immunohistochemistry method where staining brain tissue sections with specific antibodies against tight junction proteins, such as, occludin (Feldman et al., 2005; Blasig et al., 2011) and claudin-5 (Furuse et al., 1993; Hawkins and Davis, 2005) are used. These proteins are crucial for maintaining the integrity of the BBB. Disruption or loss of these proteins can indicate BBB damage or dysfunction. One crucial aspect of developing BBB microfluidic devices is the choice of appropriate cell types to construct a representative model. Several cell types have been utilized in these devices to replicate the complexity of the BBB, each contributing to specific aspects of its functionality, such as the brain microvascular endothelial cells (BMECs), astrocytes, pericytes and neurons. Here, we explore some of the commonly used cell types in BBB microfluidic models and provide references for further exploration and understanding.

Brain microvascular endothelial cells (BMECs) are one of the most important cells involved in BBB research (Pong et al., 2020). Primary human BMECs or immortalized cell lines, are commonly utilized due to their ability to develop tight junctions and establish a barrier function. BMECs line the vascular lumen while pericytes, microglia, and astrocytes form the abluminal membrane in a BBB model (Yang and Chen, 2022). These cells can be sourced from either primary cells or cell lines. Primary cells are cell populations taken directly from resected tissue samples, such as through biopsies or autopsies. Primary cells provide the most accurate results due to their high biological relevance. For example, the presence of pericytes *in vitro* plays a crucial role in promoting the development of the blood-brain barrier (BBB) characteristics. Coculturing pericytes with brain endothelial cells (BECs) enhances barrier integrity and boosts efflux transporter functions (Erickson et al., 2020). However, there are limitations of using primary cell lines for a long-term culture. Primary cells have a limited lifespan in culture, often requiring frequent isolation from fresh tissue samples. This restricts their long-term use and poses challenges for conducting extended studies or experiments over an extended period (Pan et al., 2009). They also display phenotypic variations in their characteristics leading to variability in BBB properties across different cell lines, making it challenging to establish consistent and representative models of the BBB (Rahman et al., 2016). Therefore, researchers have shifted their focus towards utilizing induced pluripotent stem cells (iPSCs) as a sustainable source of BMECs.

The use of iPSCs for studying the blood-brain barrier (BBB) *in vitro* offers several advantages and has become an important tool in BBB research (Katt et al., 2016; Pong et al., 2020; Workman and Svendsen, 2020; Lu et al., 2021). Deriving from adult cells, iPSCs are reprogrammed to a pluripotent state where they will essentially be reformed to have potential to differentiate into any cell types in the body, including BBB-specific cell types. One significant advantage of using iPS endothelial cells by Hajal *et. al*. in forming BBB microvascular network is the ability to generate personalized models that mimic native BBB ECs. These patient-specific microvascular networks can be utilized to examine barrier function and cellular structure in neurological disorders and analyze the permeability of therapeutic carriers or infectious agents. iPSCs can be derived from both healthy individuals and patients with genetically inherited neurological disorders, allowing the generation of patient-specific iBMEC models to investigate disease mechanisms and disrupted signaling pathways (Lippmann et al., 2012). For example, iPSCs have been claimed to originate from neuroepithelium rather than endothelial cells (Delsing et al., 2020) for BBB cell cultures. In order to have a biomimetic model, differentiation protocols of the iPSC-derived endothelial cells need to be optimized to increase reproducibility (Delsing et al., 2020). Since iPSCS are somatic cells that are reprogrammed, they can differentiate into all the cell types in the body. Microgravity in space could act as a stressor and cause dedifferentiation of stem cells as observed by Grigoryan and Radugina (2019). It is important that researchers know when iPSC differentiation has terminated, to make sure that the cells are at the stage necessary for the study. Protocols are in place for differentiation of iPSCs to brain endothelial cells, and factors such as retinoic acid, seeding density, and hypoxia stimulation. With this protocol, iPSC-derived endothelial cells have high TEER values, low permeability, and express tight junction proteins (Delsing et al., 2020). Before any model involving iPSC derived endothelial cells can be sent to space, the protocol for proper differentiation needs to be perfected. Recent research has shown that space microgravity has increased proliferation without disturbing the structure and function of human iPSC-cardiomyocytes (Rampoldi et al., 2022).

Traditional BBB models using 2D Transwell inserts have limitations where the transwell models have rigid surfaces that hinders direct cell-cell interactions and lack physiological mechanical forces such as shear stress, which are important for the development and maintenance of the complex *in vivo* microenvironment of the BBB. As a result, the utility and translation of 2D cultures to patient applications are constrained, as they do not fully capture the dynamic and multicellular nature of the BBB *in vivo* (Vatine et al., 2019). Tissue chip technology may provide suitable cellular microenvironments, allowing for the recreation of multicellular architectures, tissue-tissue interfaces, mechanical forces, physiochemical microenvironments, and vascular perfusion (Bhatia and Ingber, 2014). For example, Park et al. (2019) incorporated hypoxic conditions during the culturing of iPSCs to induce differentiation of cells where they produce high levels of protein and functional efflux pumps as seen *in vivo*. iPSCs offer a versatile and powerful tool for *in vitro* BBB studies. Their ability to differentiate into BBB-specific cell types, the potential for patient-specific and disease modeling, access to human BBB tissue, drug discovery applications, and mechanistic investigations make iPSCs a valuable resource for advancing our understanding of the BBB and developing new strategies for drug delivery and neurological disorder treatments.

### 3.3 Biochemical influences

BBB’s selective permeability is extremely important for the brain’s proper functioning. A compromised blood brain barrier has been seen in Alzheimer’s (Kempuraj et al., 2020), Parkinson’s (Herrera et al., 2015; Huang et al., 2020), stroke (Huang et al., 2020), traumatic brain injuries (Amoo et al., 2022), and even COVID-19 (Buzhdygan et al., 2020). In BBB dysfunction, the harmful elements of the blood can cross into the neural cells, paracellularly or transcellularly (Black, 1995). For diseases such as Alzheimer’s, Parkinson’s, and stroke, the brain promotes inflammation with the help of immune cells. The immune response of the brain involves microglia releasing small proteins called cytokines (Erickson et al., 2020). Microglia cells have a wide range of receptors for the various neurotransmitters. So, in response to synaptic activity, they can exert an impact on neuronal behavior and interaction by releasing peptides called cytokines (Augusto-Oliveira et al., 2019). Cytokines come in many flavors, with monokines, chemokines, or interleukins to name a few. They can be pro-inflammatory or anti-inflammatory in nature (Chen and Li, 2021). TNFα, IFNγ, and IL-1β are common pro-inflammatory cytokines in the brain (Ferro et al., 2021). As biochemical stimulators, cytokines are known to increase blood brain barrier permeability, especially in pathological conditions (Black, 1995). A study by de Vries et al. (1996) looked at the impact of TNF-α, IL −1 β, IL-6 on rat endothelial cells barrier integrity. They found that TEER was reduced from 100 to 150 ω-cm^2^ on exposure to the cytokines.

These cytokines and immune proteins are not the only things that contribute to BBB dysfunction. Oxidative stress due to ischemia can be an early stimulus for BBB injury. It has been linked to the activation of matrix metalloproteinase-9 (MMP-9). Brouns et al. (2011) studied the kinetics of MMP-9 as a marker for BBB dysfunction and found that there is increased MMP-9 activity in the first stage of stroke which contributes to a positive feedback loop in which MMP-9 goes on to cause greater BBB damage in the secondary phase of stroke. Underly et al. (2017) found that the same pericytes that contribute to the BBB’s stability can also cause it damage. They induce the release of MMP-9 but are also highly sensitive to it. MMP-9 acts as a tight junction cleaver, and with its activation, the group was able to see higher blood plasma leakage. Peripheral inflammation is the activation of the innate immune system with a release of proinflammatory cytokines against stimuli coming from outside the CNS (Huang et al., 2021). Researchers have found that even peripheral inflammation can cause BBB disruption. In a disease like Alzheimer’s which is characterized by the over presence of amyloid beta plaque and tau proteins, Liu et al. studied BBB permeability as a result of peripheral inflammation. They injected lipopolysaccharide (LPS) which is common inflammation inducer in the abdomen, and they saw that both chronic and acute LPS doses cause BBB disruption with a loss in tight junction proteins and allowed tau to move from the entorhinal cortex to the hippocampus in mice (Rampoldi et al., 2022). Peripheral inflammation with the combination of LPS and 1-methyl-4-phenyl-1,2,3,6 tetrahydropyridine (MPTP) was shown to cause BBB disruption in a Parkinson’s model by Garcia-Dominguez et al. (2018).

On Earth, radiotherapy is a frequently prescribed treatment for BBB tumors and vascular malformations. However, it has been shown that damages caused by the irradiation could cause damages to cells through reactive oxidative species (ROS), damaging intracellular target molecules (RobertGriffin, 2006; Allen and Limoli, 2022). Similarly, space radiation, including galactic cosmic rays and solar particle events, can impact cellular function leading to generation of ROS in BBB tissue chips, resulting in oxidative stress and damaging cellular components such as proteins, lipids and DNA integrity (Lehner et al., 2011; Fauquette et al., 2012; Allen and Limoli, 2022). Studies have shown that radiation exposure can compromise the integrity of the BBB, leading to increased permeability and potential neuroinflammation (Hart et al., 2022). Therefore, it is imperative to ensure that the development of BBB tissue chips in space would appropriate shielding to avoid radiation exposure, such as an entirely automated microfluidic system equipped with high-tech imaging systems within a box. To higher levels of radiation in space, which can affect the barrier’s integrity, inducing DNA damage and affecting cellular responses, and the overall health of the tissue chip. Understanding the effects of radiation on the BBB tissue chip is crucial for accurate interpretation of experimental results and ensuring the reliability of the model.

### 3.4 Stimulations on BBB tissue chips

Mechanical forces have been shown to provide cues for morphogenesis (Kaarj and Yoon, 2019). The tight junctions between endothelial cells are strong walls consisting of transmembrane proteins, their cytoplasmic scaffolding and associated signaling proteins, cytoskeletal filaments, and finally, membrane lipids. The tight junctions allow for paracellular transport with the pore being the highly selective path through which essential ions and water can travel, but they also have some leak permeability for larger molecules based on the breaks in between tight junction fibrils. Similarly, BBB tissue chips rely on proper fluidic and nutrient supply to maintain cell viability and function (Wu et al., 2020). Proper fluidic and nutrient supply in BBB tissue chips allows for a more accurate representation of the physiological environment to ensure the delivery of essential nutrients and maintain barrier integrity more effectively (Williams-Medina et al., 2020). However, the microgravity environment in space can affect fluid flow and nutrient distribution within tissue chips. Without the presence of gravity-driven flows, alternative mechanisms, such as passive diffusion or microfluidic-based approaches, need to be implemented to ensure proper nutrient and oxygen delivery to the cells in BBB tissue chips. As such, the designs of the tissue chips layout plays a crucial role in their functionality and the quality of experimental outcomes (Ingber, 2022), such as the two channels in the lung on a chip (Picollet-D’hahan et al., 2021; Huh et al., 2010), where nutrients are transported in the media through a separated by a porous membrane through diffusion and gravity, as well as migration of cells through cell-signaling. Similarly, BBB tissue chips can be created with multiple cell types resulting in tight junctions, replacing the porous membrane. Nutrients can be diffused, and tight junctions can be tested. However, the microgravity environment of space can have profound effects on cellular behavior, fluid dynamics, and tissue architecture. BBB tissue chips in space may experience altered cell adhesion, tight junction barrier properties, and cellular signaling differently when compared to studies on Earth due to the absence of gravity. These microgravity-induced changes can impact the functionality and integrity of the BBB model and therefore, further studies are needed to optimize the condition of BBB tissue chips before moving forward to studying BBB in space utilizing tissue chip technology (Amselem Shimon and Eyal, 2022).

In addition to microgravity stimulations affecting cell behavior, it has been shown by researchers that to form a BBB model for *in vitro* research, some fluid shear stress is essential (Cucullo et al., 2011). Partyka et al. (2017) showed that both shear stress and cyclic strain helps in the formation of tight junctions. They applied pulsatile flow and with TEER and dextran measurements, they observed pulsatile strain which is important to understand stretch transport. Jeong et al. (2018) were able to design a BBB-on-chip where they co-cultured astrocytes and endothelial cells and applied *in vivo* levels of shear stress. Their results showed formation of tight junctions as well as decreased barrier permeability. Griep et al. (2013) were able to model the BBB on a tissue chip in the presence of fluid shear stress and saw that shear stress increased barrier tightness as well as TEER values. Fluid flow in microgravity may be affected and therefore influence the functions of cells in microgravity in general. So, it is essential that a BBB chip incorporates the shear forces necessary to create mature tight junctions and to ensure proper *in vivo* modeling, as suggested by this microfluidic model by Brown et al. (2015).

Internally, the BBB is sensitive to the mechanical stress that can be induced with blood flow. For example, in a cerebrovascular bypass surgery, there is an increase in fluid shear stress that can damage the BBB and cause a hemorrhage, as seen by Wang et al. (2020). The external forces can also cause BBB disruption such as the traumatic brain injury (TBI). To understand the condition relating to TBI, Rosas-Hernandez et al. (2018) found that a 15% stretch uniaxial high-speed stretch (HSS) to a rat BBB model had low levels of cell death and some micro-tears, similar to a mild TBI (Kuriakose et al., 2018). Kuriakose et al. (2018) found that the BBB permeability increased with blast overpressure, resulting in BBB breakdowns. While these researchers have studied the effects of forces on animal models, a more cost-efficient and versatile option is to utilize a BBB-on-a-chip device that could model a TBI. A tissue chip would allow researchers to understand the effects of a TBI in terms of the resulting influx of water causing edema as well as an increased intracranial pressure (Cash and Theus, 2020).

### 3.5 Tissue chips fabrication techniques and design layouts

Microfluidic devices have revolutionized the field of biomedical research and diagnostics by offering precise control and manipulation of fluids on a small scale. These devices are commonly used to study biological phenomena, perform lab-on-a-chip analyses, and facilitate drug discovery. The fabrication of microfluidic devices involves various techniques that allow for the creation of intricate channels and chambers, enabling precise fluid flow and manipulation. Several fabrication methods, such as photolithography, soft lithography, stereolithography (SLA) (Qin et al., 2010), and bioprinting are utilized to create these organ-on-a-chip devices, each offering unique advantages. Photolithography is a commonly used technique in microfabrication to create desired patterns onto a substrate using photosensitive materials with light through a mask, seen in Figure 2, where the pattern would develop and, etch onto the exposed regions. While photolithography is popular, it requires high setup cost and time, and is not flexible enough to respond to changes required (Alessio Bucciarelli et al., 2022). Soft lithography is a popular fabrication technique used in the production of microfluidic devices. It involves creating elastomeric chips by using a rigid master mold. Typically, a material such as Poly (dimethylsiloxane) (PDMS) is poured onto the master mold, allowing it to replicate the mold’s features when cured. Soft lithography offers advantages such as simplicity, low cost, and the ability to produce flexible and biocompatible microfluidic devices, making it a widely adopted method in the field (Haring et al., 2017).

**FIGURE 3:**
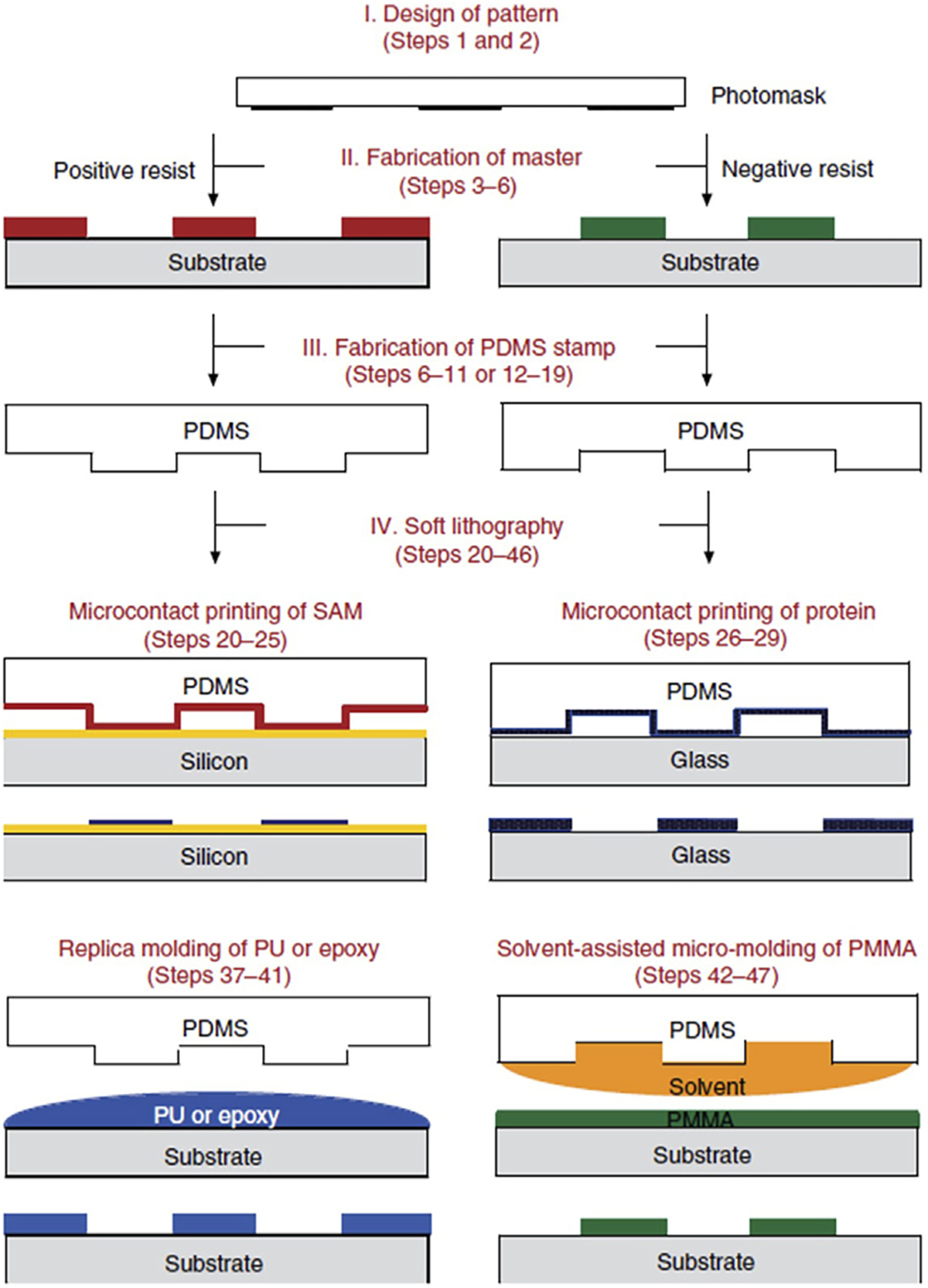
Schematic illustration of the four major steps involved in soft lithography and three major soft lithographic techniques (Cash and Theus, 2020) [reproduced from Qin et al. (2010), Nature Protocols, with permission from Springer Nature].

3D printing, particularly fused deposition modeling (FDM), is another prevalent technique for fabricating tissue chips, allowing the layer-by-layer deposition of biocompatible materials to create complex tissue structures (Kristiawan et al., 2011; Mazzanti et al., 2019; Salman et al., 2020). SLA, seen in Figure 3, is commonly used in various industries for rapid prototyping and additive manufacturing of intricate and detailed 3D models in applications such as product design, automotive, aerospace, healthcare, and jewelry. SLA allows for the production of prototypes, functional parts, and even custom-made products where it uses a specific type of 3D printing technique requiring laser to cure liquid photopolymer resins and build three-dimensional objects layer by layer (Shirvan et al., 2021). It is one of the earliest and most widely used 3D printing methods, offering high resolution and the ability to create complex microfluidic channels and structures. In one of the studies, higher throughput methods were developed to overcome the limitations of both photolithography and soft lithography (Alessio Bucciarelli et al., 2022). Unlike SLA, Digital light processing (DLP) uses UV projector for a more efficient fabrication speed, was developed to produce microfluidic devices for biomedical applications with high aspect ratio and resolution to overcome the limitations of soft lithography (Alessio Bucciarelli et al., 2022). Bioprinting enables precise deposition of living cells and biomaterials, offering the potential for creating 3D structures with soft tissue properties (Tarassoli et al., 2018; Soumitra Das and Basu, 2019). Bioprinting and tissue chip technologies can be combined to generate realistic organ models, as demonstrated by the work of Silvani et al. (2021), who integrated soft lithography and bioprinting to study glioblastomas and their impact on vasculature. Their successful design incorporated a microfluidic model with independent compartments, where a tumor construct was directly bioprinted using a fibrin hydrogel. They explored the effects of simulated microgravity on glioblastoma functionality, highlighting the importance of studying the blood-brain barrier and microgravity effects. These fabrication methods hold promise for advancing therapies and treatments for diseases on Earth and in space exploration, offering enhanced control and the ability to create intricate designs.

The design of a microfluidic tissue chip often depends on its applications. However, the primary goal of microfluidics is to achieve precise and accurate manipulation of fluids while minimizing the usage of reagents and equipment (Low and Tagle, 2017). A basic design for tissue chips contains microchannels with inlets, outlets, and a perfusion channel or membrane and chamber where the interaction between cells and reagent occurs. The simplest design is the straight channel design where channels are straight and parallel to each other, often used for simple fluid transport applications. The flow of fluids inside the microchannels is regulated by the valves or by the speed of input and output. In a typical tissue chip, the channels are often designed to be parallel to each other horizontally (Pattanayak et al., 2021). However, for the BBB, endothelial cells in the upper layer and the brain cells are grown in the lower layer. These channels are parallel vertically, separated by a porous membrane which is what acts BBB (Pattanayak et al., 2021) for the ease of integration of different cell types and tissues. For example, applied cyclic radial strain by using a dual chamber design, filling the top and bottom chambers with media and endothelial cells, respectively (Jin et al., 2020), then relied on gravity to diffuse down towards the bottom chamber. However, the layout of tissue chips used in space should be designed to accommodate the inclusion of different conditions and protection from space radiation and vibration from the launch, with regards to weight and sizes imposed by space missions. Optimizing the layout allows for the efficient use of space and resources available on spacecraft, ensuring compatibility with the specific requirements and limitations of the mission. Proper and standard layout design also facilitates scalability, enabling the adaptation of tissue chip systems to larger dimensions or the incorporation of additional functionalities without compromising the overall performance. Designs of tissue chips for cell culture are highly customizable and can be adapted to model different tissues and organs. The goal is to create a system that mimics the structure and function of human tissues, enabling researchers to study disease mechanisms and drug responses in a more accurate and efficient manner.

Understanding the impact of these factors on tissue chips is essential for the accurate representation of the BBB on a tissue chip. The human body is characterized by a complex interplay of various influences that contribute to the proper functioning and efficiency of cells. When developing an *in vitro* model of a specific body part, it is crucial to incorporate as many relevant factors as possible to facilitate the application of the obtained results. Achieving a biomimetic model ensures the reliability and reproducibility of the experimental outcomes. For instance, in the context of drug delivery, a simplified BBB model may allow a drug to permeate across the barrier. However, in clinical trials, the drug may face challenges such as enzymatic degradation or immune system responses. Likewise, the drug carrier might demonstrate adequate diffusion in a simplified model, but its behavior could significantly differ under mechanical stress, leading to burst release. By incorporating these complex influences into a tissue chip model, researchers can study the impact of these factors and obtain more comprehensive and realistic results. Comprehending the influence of various factors on tissue chips is vital to create an accurate representation of the BBB to develop a biomimetic model that incorporates these influences allows for reliable and reproducible results (Marino et al., 2018). Such a model can provide valuable insights both on Earth and in space, where microgravity introduces additional effects, enabling a comprehensive understanding of the BBB’s behavior and its response to therapeutic interventions.

As noted from the variety of layouts and designs, tissue chips are complex for researchers here on land. The difficulties of bringing these tissue chips for study in space brings with it its own set of challenges. One of the challenging issues the microfluidic devices will face is surviving the launch to the ISS. All tissue chips in the Tissue Chips in Space initiative have to be adapted to face the shocks of launch and return, and also, all of the machinery, including tubing, incubators, and even microscopes have to be in miniaturized systems so that they can help provide meaningful results, while maximizing results collection (Low and Giulianotti, 2019). Based on phase I development results, specimens printed aboard the ISS have presented different mechanical properties where microgravity does not have engineering significant effects on manufacturing processes (Prater et al., 2019). This work is powerful as it gives us the opportunity to pursue BBB research on the ISS by starting off with 3D printing microfluidic models with systems already connected on orbit preventing tissue chips from facing the launch forces.

## 4 Current BBB tissue chips on Earth

### 4.1 The positive impacts of current BBB tissue chips

There are many 2D cell culture systems in place to study the human nervous system. However, there are a few microfluidic devices readily developed for study on Earth, but fewer devices have been sent to the International Space Station (ISS) to study the effect of microgravity on BBB. The work on flight hardwares in the past decades has been helpful in the development of the BBB model for space research. In the following sections, the advantages and limitations of BBB on Earth are discussed and what it means to send BBB tissue chips in space. Advantages of tissue chips such as one designed by Cho et al. (2017) created a self-assembling BBB spheroid that includes different cell types to create tight junctions within. The spheroids are composed of a core of astrocytes and endothelial cells as the shell where they were able to see expressions of tight junction proteins, some even more prominent than a conventional Transwell model due to its self-assembling capabilities. Rather than doing a TEER analysis, they replaced the permeability assay with a test using dextran through the membrane. This way they were able to analyze images obtained using the dextran for their permeability testings. The group was able to see that their spheroids maintain barrier integrity as no high weight dextran was able to pass through. While this model could prove useful for drug screening and other Central Nervous System (CNS) therapeutics analysis, the model cannot fully be biomimetic as it lacks the dynamic fluid flow that is normally present.

The BBB plays an essential role in protecting the brain from harmful substances and maintaining the brain’s stable environment, like invaders like nanoscale particulates. Studies have shown that spaceflight can also affect the BBB. In 2021, Kim et al. (2021) published a study where they created a human neurovascular unit (hNVU) out of PDMS to understand how the BBB is influenced and infiltration of microbial pathogens. Although their design was vertical, it could still function horizontally. One main advantage of their chip is the vertical design of the chip. This allows for better spatial utilization, and there is passive trapping of the fungi in the cell layer. The chip’s ability to be used both horizontally and vertically adds to its versatility and gives the chip an advantage if it were to be manufactured for large scale use. The stacking of tissue chips vertically could allow researchers to take on an organ or a multiorgan study, as this group showed with the liver as an organ system in their tissue chip. This is a powerful advantage as most processes in the body are collaborations between organ systems. Utilizing the multi-tissue chip technology, researchers could determine the absorption, distribution, metabolism, and excretion (ADME) process during screenings of compounds and can observe effects of drugs in certain tissue chips in the process and rely less on animal models. In this case, the group was also able to incorporate paracrine signaling, and studying what factors or secretions are observed acted to boost integrity of the model.

A true in vivo model would need to account for many other cell types such as astrocytes and pericytes which play an important role in the BBB’s structure. As seen in studies by Lyu et al. (2021), their research on the evaluation of the restorative potential of stem cell therapies for ischaemic stroke can be observed in their tissue chips. In the study, their microfluidic model was multi-channeled, allowing for the inclusion of different cell types to be positioned next to each other within the same focal plane which can be observed in the microscope. They utilized their tissue chip to observe how stroke affects the BBB, and to further understand the restorative properties of stem cells. Their 3-channel chip contains the blood channel where the bloodstream can be mimicked, the brain channel where the human neural cells were embedded in a hydrogel matrix, and finally, a cerebrospinal fluid (CSF) channel. They noted that the presence of CSF substances would validate the biomimetic nature of their design where they can form proinflammatory substances from the blood-mimicking channel. While the BBB is the interface that is commonly studied, the brain-CSF barrier is just as important when studying the effects of therapy, especially for any future directions in drug delivery. The direction of current tissue chip technology by Zhonglin’s group could be proven to be useful in space as it would allow a thorough analysis in disease modeling or drug screening. Knowing how a drug affects blood, brain, and then entering into CSF, would give a more accurate *in vivo* analysis in space.

**FIGURE 4:**
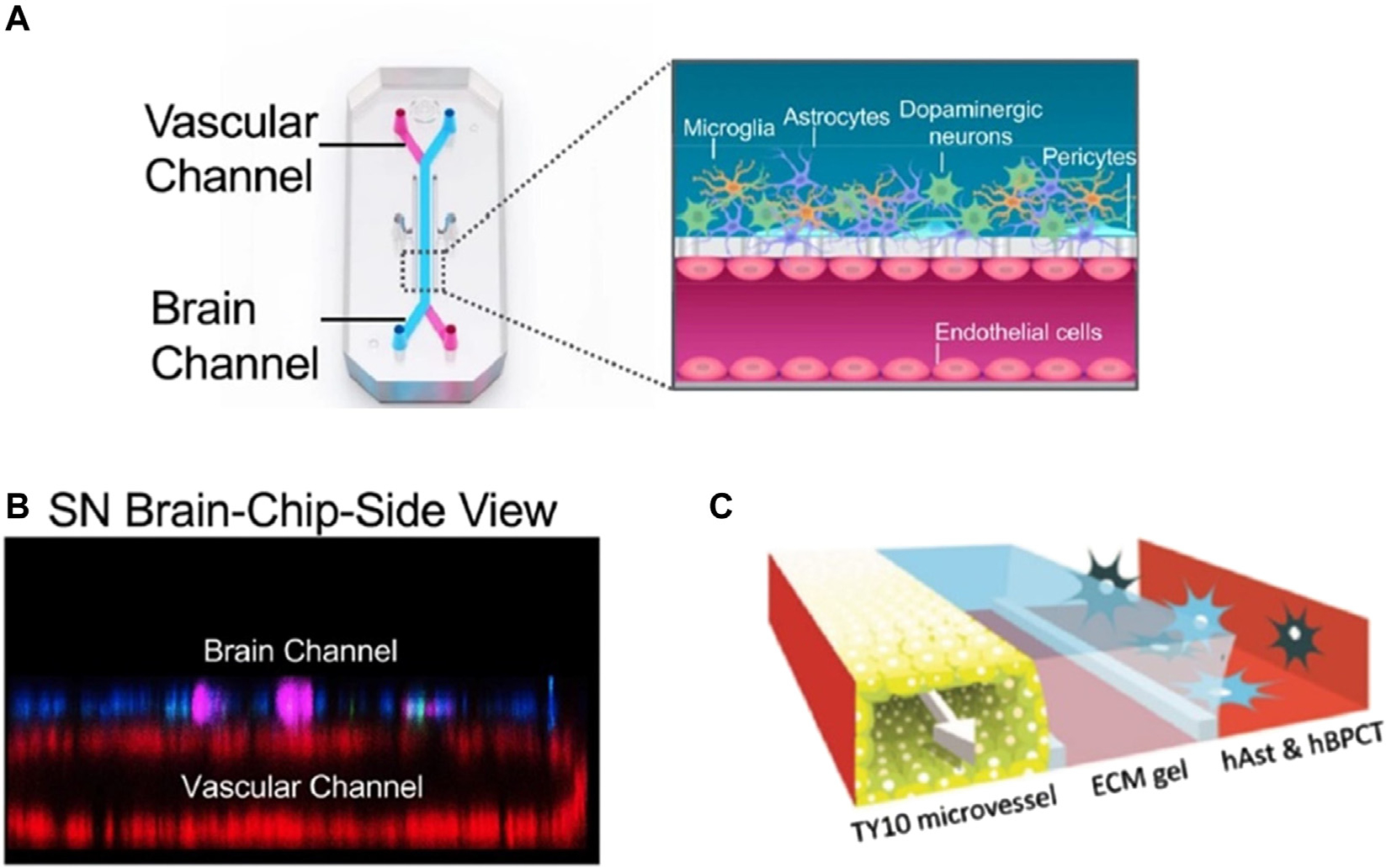
Pediaditakis et. al. shows a diagram of their BBB tissue chip, with a schematic of the types of cells in each channel. Panel **(A)** shows the schematic diagram of the tissue chip used in their research, with the cross section of the multi-cell type used in the chip [reproduced from Pediaditakis et al. (2021) licensed CC-BY-4.0]. Panel **(B)** shows the SN Brain-chip confocal side view of the tissue chip itself (Hinojosa, 2017) [reproduced from Pediaditakis et al. (2021) licensed CC-BY-4.0]. Panel **(C)** shows different BBB models to recapitulate the BBB system where porous membrane is used (Seo et al., 2020; Wevers et al., 2018) [reproduced from Wevers et al. (2018) licensed CC-BY-4.0]. This shows that a standardized tissue or organ on a chip model may be needed in order to incorporate a standard flight hardware system in space to provide an advantage for pursuing any further research in space.

As research begins to move away from 2D systems and Transwells, and the need to understand the mechanisms of the brain grows, many researchers have started coming up with their own 3D designs to model the BBB. Recently, In 2021, Iosif Pediaditakis and his team published a paper where they created a tissue chip system to model Parkinson’s disease to understand how the pathology compromised the blood brain barrier. Figure 4 represents the tissue chips used by Iosif et al., designed to be compatible with Zoe Culture Module. This is a module created by Emulate Inc. and is compatible with all of their brain-on-a-chip designs. Emulate Inc. received a grant from NiH and sent their designs to the ISS for further study in 2017 (Hinojosa, 2017). Although Pediaditakis et al. (2021)’s chip was compatible with Emulate Inc.’s products, the success rate of the incorporation of flight hardware with Zoe Culture model is still unknown. Table 1 shows the limitations and advantages of current BBB tissue chips on Earth.

### 4.2 Limitations of current research in BBB tissue chips

On Earth, there has been a lack of relevant cellular models to help understand the progression of Parkinson’s disease (Martinez-Morales and Liste, 2012). In a clinical setting, Parkinson’s disease in a patient can be diagnosed based on different muscle movement, or lack thereof, such as resting tremors, bradykinesia or fixed facial expressions (Fitzpatrick et al., 2009). However, it is challenging to observe and predict the disease behavior *in vitro*. Growing cells on Earth to model Parkinson’s disease *in vitro* seems to be inefficient as one would have to let cells mature to exhibit Parkinson’s biomarkers. *In vitro* studies have been essential in advancing our understanding of the disease mechanisms and testing potential treatments. However, there are several limitations to *in vitro* studies of Parkinson’s disease, like lack of complexity of the *in vitro* model, limited access to human brain tissue and lack of systemic interaction such as the immune system’s role in the progression of PD such as immune. However, the researchers from the National Stem Cell Foundation (NSF) have studied neuroinflammation pathways diseases such as Parkinson’s and multiple sclerosis by observing 3D organoids under microgravity conditions on the ISS. The models remained in space for a month, and they were able to observe differences in gene expression and protein secretion. Although the results have not been published, it shows that we are heading in the right direction to develop an *in vitro* system that can help test potential therapies for the disease (Andres Bratt, 2019).

To overcome the limitations of cell culture faced on Earth, culturing cells in space may accelerate the process of aging where there is a decrease in cell proliferation due to the lack of gravity (Wnorowski et al., 2019). An increase in cell apoptosis is observed when studying cell culture in microgravity (Madelyn Arzt et al., 2022). On Earth, this model by Pediaditakis et al. (2021) allows for sampling of effluent channels from the outlets at various time points in addition to the transparency of the tissue chips allowing for imaging. Unfortunately, like many other models previously described, this tissue chip model would be a challenge to model in space. Due to the complexity of BBB, the development of tissue chips that accurately mimic the complexity of human organs and tissues remains a challenge. Recreating the intricate cellular architecture, vascular networks, and mechanical forces within a small microfluidic platform is a complex task. Scaling up these models to larger, more physiologically relevant sizes without compromising functionality is also a challenge. The cost to develop tissue chip models in space may increase exponentially as the scale of tissue chips increases. Variations in cell sources, culture conditions, fabrication techniques, and experimental protocols can lead to inconsistent outcomes. Because each design requires a different setup, it presents various technical challenges due to specialized equipment and technology for their development and maintenance and therefore increasing the cost of research. A standardized model could be useful when utilized in space where equipment to set up the microfluidic system can be reused for multiple experiments.

Many tissue chip studies on Earth have been proven successful when studied on Earth. For example, a study done by Yan Li et al. looked at the impact of indoor airborne nanoparticles on the blood brain barrier. One advantage in Li et al. (2020)’s tissue chip design is that they accounted for pressure differences, akin to TEER measurements in monolayer cell cultures In a BBB *in vivo* condition, pressure differences are typically measured between the blood and brain sides. Normal intracranial pressure in the brain is within the range of 5–15 mmHg (Rangel-Castilla et al., 2008), while pressure in a blood vessel varies based on systolic or diastolic conditions. Accounting for this pressure difference makes this chip even more similar to an *in vivo* model. The more factors present in the body researchers can mimic, the greater precision their data will have. This study could provide some insights as to how the cells may act in tissue chips when performed in space where the different mechanisms can be observed under microgravity.

There are multiple issues that may arise when imaging BBB tissue chips over time on Earth or in space. On Earth, technicians may examine the tissue chip at any point in time, which may not be the case when performed in space (Lyu et al., 2021). Performing TEER measurements in tissue chips for space studies is still proven to be a challenge because culturing cells in general requires significant resources including funding, time, and collaborations among researchers and implementation partners which could also impede the development of standardized protocols and guidelines (Piergiovanni et al., 2021). Researchers would need to ensure that the measurements are conducted under controlled conditions to minimize any potential confounding factors related to microgravity or other space-related factors. This may involve developing specialized and automated equipment and advanced protocols that can maintain a stable environment for the tissue chips. Furthermore, with the rapidly evolving technology, the dynamic nature of tissue chip technology makes it difficult to develop standardized protocols and guidelines that can keep up with the evolving technology.

Overall, the effects of spaceflight on the BBB are still not well understood, and further research is needed to fully understand the potential risks to astronauts’ brain health during space missions. One of the limitations in many of the BBB tissue chip designs on Earth is they are not a complete representation of BBB. Because the BBB is a complex network, and the barrier is present wherever there is an interface between brain cells and blood vessels, there would be a large blood supply leading to the brain. Hence, the tissue chip design by Lyu et al. (2021)’s group may not be the full representation of a brain due to their small surface area. The small surface area of their BBB in the tissue chips can be akin to small sample size where it requires much effort to maintain and perform which defeats the purpose of the tissue chip development. Scaling the fabrication and setup of the tissue chip to represent a native and vital organ is still proven to be a challenge for researchers both on Earth and in space. Similarly, TEER measurements in tissue chips have been developed for use on Earth. However, it has been a challenge to incorporate TEER readings into tissue chips for space research into space flight hardware. This is possibly due to the difficulty observing the interactions across vertical layers through imaging and the height difference of channels in tissue chips could lead to limitation in observing the cell-cell-interactions. Addressing these limitations requires interdisciplinary collaborations, advancements in bioengineering, improvements in cell culture techniques, and standardization efforts. The increasing cost of conventional prototypes for manufacturing tissue chips and within clean room facilities, requires the need for a more affordable alternative (Rodriguez et al., 2023). Continual innovation and refinement of tissue chip technologies will pave the way for more accurate, reliable, and clinically relevant models for studying human biology and disease.

## 5 Biosensors incorporated into a BBB tissue chip

Real-time monitoring from any studies, especially in the tissue chips are imperative to fully understand the cell-cell interaction, influences of a long-term cell culture, or effects of microgravity to specific cell types, and overall mechanisms of cell activities. This includes data about viability of cells, metabolic activity of the constructs, or on pressure and permeability (Kilic et al., 2018). Prior to incorporating biosensors in tissue chips, high-performance liquid chromatography/mass spectrometry (HPLC/MS) or multiplexed (bead-based) protein-binding/DNA-binding assays were the most practical and commonly used techniques for monitoring tissue chip samples. These analytical methods are powerful because their measurements offer information on many biomarkers in a cellular response all at once. However, not only they can only serve as end-point analyses, but there can also be interference due to the components of the serum that are flooded into the chip. Furthermore, the cells could be easily damaged while processing tissue chips to collect samples to do protein and gene analyses, histology, or viability assays.

Sensors in tissue chips can give information on not only the physical factors such as stimulations, pressure, but also on biochemical parameters. This real-time data collection is essential for various applications, especially in a drug testing procedure where drugs can either cause instantaneous or delayed reactions on patients’ personalized tissue chips. Both are important to study, but some can only be detected during an on-going study based off of continuous data collection. One important data point that researchers are interested in is the amount of oxygen present in the vasculature (Leung et al., 2022). Oxygen is one of the most important molecules that must cross the blood brain barrier for the proper functioning and metabolic activity of neurons. The loss of oxygen may severely compromise the system, especially in a long-term mission.

A study by Schneider et al. (2022) integrated two major components to form a simplified cardiac u-tissue generation by adding an automated electrical stimulation to the chip when needed, as well as oxygen sensors in the chip. The oxygen sensor detection dye is in a resin which they were able to mold with their PDMS chip design. The oxygen sensors were positioned below the tissue chambers, which allowed for direct contact to the tissues and increased data accuracy. This group was also able to add electrical stimulation capabilities without the integration of complex electrodes. Stainless steel fluid connectors in the media layer of their design acted as stimulators. Based on their methods, the electrical field strength was simulated using finite elements and pacing of tissues were field-paced with custom-built Arduino-based electrical stimulators to establish beating rates of the cardiac tissues based on pacing frequencies of stimulations. This study has shown the group have successfully provided a tissue chip where assessing metabolic tissue activity such as oxygen level in the tissue chip under influence of external pacing with electrical stimulation is possible. Similarly, tissue chips developed by Ortega et al. (2019) allowed the researchers to give stimulation as well as measure cytokine release from muscle simultaneously. They used indium tin oxide (ITO)-interdigitated arrays (IDA) electrodes for electrical stimulation, or lipopolysaccharide (LPS) solutions for biological stimulation. The measurement of secretions was done using high-sensitive screen-printed gold electrodes and antibodies. An advantage of this design was that they included both electrical and biological stimulations in their design, allowing them to obtain information on cytokine release from the same chip. By functionalizing the electrodes with cytokine specific antibodies, the researchers created a selective system that could sort out the cytokines. In both studies, their microfluidic systems allow for various tissue engineering studies in the tissue chips with integration of external stimuli which could prove to be beneficial to application in space studies of tissue chips.

Current models with integrated biosensors measuring oxygen, pH or glucose content relying on fluorescent tags. However, they often rely on the availability of fluorescent tags which could prove to risk disturbing the microfluidic systems. The newer models are coming out with antibody-based electrochemical sensors in tissue chips where real-time monitoring is based on biochemical signals released by the cells in the tissue chips (Morales and Aleman, 2016; Ortega et al., 2019). Cytokines are secretions of immune cells that go on to affect the behavior of other cells (Zhang and An, 2007). Astrocytes and microglia found in the brain release these proinflammatory cytokines in the BBB. When studying an infectious disease, understanding and detecting cytokines in real time could aid researchers in understanding the BBB’s immune response. Cognetti et al., in collaboration Miller’s and McGrath’s group (Ajalik et al., 2022) developed a microfluidic device with two channels, the top carrying the media, and the bottom was used by the sensor to monitor cellular secretions. Mimicking systems like TEERs in 2D cell culture, they successfully tested their barrier system to test for tight junction-disrupting peptides (TJDPs) in their 3D cell culture devices. In between the two channels of their device, cells were cultured on nanoporous silicon nitride membranes. Cells in the device will secrete cytokines to the bottom channel and an activating media was flown through the top channel to measure secretion level. Since the bottom channel’s inlet and outlet had been blocked, the cytokines would diffuse down towards the photonic sensor chip at the very bottom. The researchers were able to observe the crossing of big molecules such as the pentamer in the devices with the sensors developed in the group. By doing these tests, the researchers were able to capture data on not only cytokine secretion but also on the functionality of the barrier in the tissue chips (Cognetti et al., 2023). They utilized diffusion of the analytes to power their measurement mechanisms. This device has future applications with other intruders to the barriers, including microbes. The ability of this device to observe large molecules and their interactions with the barrier is especially important in order to study vehicles for drug delivery. Having a physical barrier made of nanoporous silicon membranes makes this design more biomimetic. The advancement of sensing cytokines with antibody-based sensor technology is a promising approach to the field of drug discovery as well as space research.

Many research groups have designed and developed various devices to study the BBB. However, only a few have investigated monitoring the barrier functions in microfluidic platforms. The use of the TEER system in the *in vitro* studies has yet to be translated to the tissue chip platform, until 2017, a study by Henry et al. (2017) a TEER monitoring chip was designed and fabricated with a goal to non-invasively test virtually any barrier. The TEER values were detected utilizing two electrodes to stimulate and two to measure the voltage difference. They found that the TEER values they obtained can reach 1,700 Ω, which falls within bounds of an *in vivo* BBB. The TEER measurement is important to prove the validity of the barrier. A low TEER value suggests that the barrier is not as intact as *in vivo* conditions. Their use of separate electrodes to stimulate and detect seems like a good idea. Since TEER is a measure of resistance, basic calculations can be done to convert voltage to a resistance with Ohms law. While this is a powerful measure, even a small hole in this barrier could cause drastic changes in measurements (Cognetti et al., 2023). To account for this, the team could use an electrode array to obtain measurements to get representative data of the whole barrier rather than one section alone.

A BBB tissue chip that could incorporate a multi-sensor unit would be optimal for a long-term research application in space. A design that incorporates cytokine sensors to analyze real-time activity of the BBB tissue chip, while maintaining viable barrier functions and cell viability, including the responses to basal culture conditions. A multisensory unit, one that monitors metabolite activity, uptake and secretion, and TEER level sensing will provide the scientists on Earth and in space with enough information for long-term missions. Since the sensors can be built into the chip, as mentioned in the previous examples, it would be helpful for both researchers and astronauts when analyzing the samples in tissue chips in a long-term mission. Sensors and TEER systems have been proven to be challenging to be incorporated into the space flight system. Therefore, one of the goals for both engineers and researchers is to accommodate the incorporation of sensors and TEER so that they fit into the space flight system. If the electrodes are incorporated inside the tissue chip, TEER has the potential to give real-time recordings. Demonstrating the relevance and predictive capability of tissue chip models compared to *in vivo* systems and clinical outcomes is essential. Ensuring that the data from biosensors obtained from tissue chips can be effectively translated to clinical decision-making is an ongoing challenge.

Biosensors are devices that can detect biological signals and convert them into electrical or optical signals. In space, biosensors can be used to monitor the status of biological experiments in real time to detect and measure the presence of chemical signals and its changes. The addition of biosensors to detect biochemical changes or pressure changes of the endothelial and epithelial tissue activities would be favorable for research application (Yau et al., 2023), but there are still many shortcomings when it comes to the utilization of biosensors, especially during long duration space flight missions. One of the challenges would appear only in a long-term mission where the media in the tissue chip may turn cloudy due to cells secreting various chemicals into the system, decreasing their sensitivity or accuracy. In addition, accuracy or sensitivity of a biosensor often depends on the stability of the platform they are incorporated in. Therefore, biosensors incorporated in tissue chips to be sent to space will experience the rigors of launch and strong vibration that could compromise the sensitivity of biosensors. At the same time, although many have started incorporating biosensors into tissue chips to study the function of cells, tissues, and organs in microgravity, many have yet to incorporate the disruption of space radiation to the organs chip in terms of a long-term exposure of more than 1 month (Mu et al., 2022). Biosensors are an important tool for long-term cell culture in space, providing a way to monitor and optimize cell growth and behavior in real-time. The use of BBB tissue chips in space may not be optimized but current advances in tissue chip technology on Earth will improve the functionality of tissue chips in space which in turn may benefit the study of human health in space in a long-term mission of more than 6 months.

## 6 In-space BBB tissue chips

A journey in space is incredibly demanding even for the best of humanity. This is because the human bodies tend to adjust unnaturally when subjected to different environments. Acceleration of disease progression such as bone and musculoskeletal system losses have been observed in extended microgravity (CatherineYeung et al., 2020). In 2016, NCATS collaborated with the ISS National Lab to utilize tissue chip technology to achieve the goal of studying health concerns by studying biomarkers, bioavailability, efficacy, and toxicity of therapeutic drugs during space travel (Mu et al., 2022). Since then, various organ-on-a-chips developed by different research groups have been flown to the ISS to study the effects of microgravity to the organ chips. Organ chips such as muscle-on-a-chip (Smith, 2018), heart-on-a-chip, kidney-on-a-chip by Himmelfarb and Brigham and Women’s Hospital were all developed to model human diseases that mimic the physiology and pathology of major human organs and tissues.

Space travel was also shown to negatively impact human physiology and health. Researchers found that spaceflight could impair human eyesight which could last for a long time (Mader et al., 2011). In 2020, the term Space flight Associated Neuroocular syndrome (SANS) was coined to describe the findings that occurred on the astronauts during short and long duration space flight. A study by Professor Kramer et al. (2012) highlights the lack of gravity can cause changes in visual acuities which can lead to a phenomenon akin to idiopathic intracranial hypertension occurring on Earth. The study demonstrated the impact of space flight on human eyesight, but the interaction between the human nervous system and the brain under microgravity is still largely unknown (CatherineYeung et al., 2020). Microgravity causes the brain to swell and an increase of cerebrospinal fluid, which surrounds the brain and spinal cord leading to impaired eyesight (Lee et al., 2020). Therefore, it is impertinent to explore the relationship between these two before a long-term mission. Interestingly, Hinojosa groups have sent their BBB tissue chips to the ISS not only to access their newly developed automated hardware in space, but to observe real-time cell-cell interaction through different automated techniques such as imaging as they have mentioned. However, no results have been obtained since the project end date of February 2019. The success in understanding the mechanism and interaction between the brain and central nervous system as well as the role of BBB would provide insights into drug discovery both in space and on Earth.

The latest BBB tissue chips studying neuropsychiatric drug screening sent to space were awarded to Professor Leong from the Columbia University Health Sciences funded by NIH NCATS Tissue Chips (Leong, 2018). Their research combined cerebral organoids (CO) with tissue-engineered blood vessels and BBB forming a microfluidic device to understand the relationship between the nervous system and vascular system. Due to inability to accurately translate *in vivo* data and limited access to human brains, it is a challenge to study the interaction of drugs with human brain tissues. Utilizing the BBB chip developed in Professor Leong’s group, they would be able to screen various neuropsychiatric drugs and treatment strategies on different diseases. Studying the system in space may provide an advantage on modeling diseases that may not be grown on Earth. Another microphysiological system that was studied in microgravity was developed by Hinojosa *et al*. in 2017. Their BBB platforms were used to study the effects of microgravity on BBB in both healthy and diseased conditions. Drug screening on Earth has been a challenge, but the study of drug screening in space has added an extra variable to the study of drug interaction with cells due to the abnormal conditions. However, the results of drug screenings in space may be beneficial when translated to the studies of therapeutic fields on Earth. Therefore, the inclusion of biosensors in tissue chips may expedite the field of therapeutics in the near future.

While therapies are getting increasingly advanced, many are still suffering. Standardizing tissue chips entails the development of uniform protocols, guidelines, and quality control procedures to promote reproducibility and enable meaningful comparisons between various tissue chip models. Tissue chips allow us to create disease models to understand mechanisms and conduct drug screening on potential treatments. However, the standardization of BBB tissue chip models may need to be developed before further applications of the applications of BBB tissue chips in space can have various benefits including advancing our understanding of the BBB’s complex physiology, developing new therapeutics for neurological diseases. Researchers can further understand the effects of microgravity and space radiation on the BBB’s structure and function. By sending these research tools to space, we can utilize microgravity to gain a deeper understanding and potentially devise new treatment outcomes. While a few BBB tissue chips have been fabricated and studied, biosensors could provide real-time monitoring and bring researchers much closer to lasting therapies.

## 7 Conclusion

While 2D monolayer cell cultures have been utilized for a long time, tissue chips have started gaining traction over the past few decades, taking over much *in vitro* research due to their highly biomimetic results, high throughput, incorporation of fluid flow, as well as decreased need for animal models. Many have utilized tissue chip technology to mimic different organ systems such as the BBB where the researchers would study drug delivery and disease modeling. Although the TEER system in a monolayer cell culture is a common system to mimic BBB *in vitro*, it is worth taking note that the technique does not accurately represent a whole system due to their flat surfaces. BBB tissue chips are affected by various biological, chemical, and physical factors on Earth, performing experiments in space can introduce additional factors such as microgravity, radiation and temperature fluctuations affecting the microfluidic systems. Therefore, the use of tissue chips with the TEER system is one way to represent the BBB physiological system more accurately than tissue culture plates. Microfluidic systems offer precise control over fluid flow, enabling the manipulation and analysis of cells in a highly controlled environment. However, the complexity of recreating physiological conditions and scaling up these systems for large-scale cell manufacturing or high-throughput screening has posed significant obstacles. Nonetheless, ongoing research and technological advancements hold promise for overcoming these challenges, paving the way for the realization of such endeavors in the near future. On Earth, BBB tissue chip models have started incorporating other external factors such as TEER measurements, types of biomaterials, manufacturing techniques like SLA, as well as biomechanical stimuli to make their results more applicable and closer to *in vivo* condition. Overall, the effects of spaceflight on the BBB are still not well understood, and further research is needed to fully understand the potential risks to astronauts’ brain health during space missions. Now, as researchers have turned to study human health in space, BBB tissue chips can be a tool to understand function mechanisms but also for therapeutics by using microgravity as a tool for ease of use and to understand the effects of low gravity in itself.

## Figures and Tables

**TABLE 1:** A few examples of the limitations and advantages of a few research groups developing BBB tissue chips. Currently, Pediaditakis et al. (2021) developed a BBB platform that allows for various cell types such as dopaminergic neurons, astrocytes, microglia, and pericytes in the brain channel and BMECs in the vascular channel. However, their system did not account for the pressure that may influence the growth of the cells as with other research groups such as Jin et at. (2020). Li et al. (2020) on the other hand account for pressure difference in their BBB tissue chips but they only looked at a couple cell types in their BBB tissue chips. Accounting for pressure differences and including various cell types in a BBB tissue chip can help researchers to observe the cell-cell, tissue-tissue and organ-organ interactions. One common point in all the designed tissue chips are that they can be imaged real-time, which is a major advantage.

Research	Advantages	Limitations
Modeling alphasynuclein pathology in a human bramchip to assess BBB disruption	• Accounted for many cell types	• Pressure not accounted
Pediaditakis et al. (2021)	• Allowed cell-cell interactions	• Lack of Immune cells
	• Tested for tight junction expression	
	• Allows for imaging	
	• Allowslowsfor sampling from the effluent channels	
	• Continuous perfusion and fluid flow	
Study of the neurotoxicit: of indoor airborne narsoparticles based on a 3D human BBB chip	• Accounted the Pressure difference by having different volumes of media	• Only looked at 2 cell types (astrocres and HUVECs). So there was a lack of cell-cell interaction
Li et al. (2020)	• Constant fluid flow	• No microglia
	• Cells are fluorescently labeled for ease of imaging	
Fungal brain infection modeled in a human neurovascular-usut-on-a-c hip with a functional BBB	• Vertical and horizontal chip design	• No pressure differences
Jin et at. (2020)	• Incorporated passerine signaling using an Immunoassay for human angiogeneus factors	• Only used ECs. Perigees and astronytes. No mscroglia
	• No individual perfusion tubes	
	• Have used this design m a molt organ chip study—they have showed ns relevance	
	• Allowed for imaging	
	• Tested for tight junction expression	

## Nomenclature

2D: Two-dimensional
3D: Three-dimensional
BBB: Blood brain barrier
NCATS: National center for advancing translational sciences
ISS: International Space Station
NASA: National Aeronautics and Space Administration
TEER: Trans-endothelial electrical resistance
FDA: Federal Drug Administration
ROS: Reactive oxidative species
CNT: Carbon nanotube
JBNt: Janus base nanotube
JBNp: Janus base nanopiece
JBNm: Janus base nanomatrix
EC: Endothelial cells
CNS: Central nervous system
CAD: Computer aided design
PMMA: Poly (methyl methacrylate)
PDMS: Polydimethylsiloxane
PVA: Poly-vinyl alcohol
